# A Visual Data Mining Tool that Facilitates Reconstruction of Transcription Regulatory Networks

**DOI:** 10.1371/journal.pone.0001717

**Published:** 2008-03-05

**Authors:** Daniel C. Jupiter, Vincent VanBuren

**Affiliations:** Department of Systems Biology and Translational Medicine, Texas A&M Health Science Center College of Medicine, Temple, Texas, United States of America; University of Glasgow, United Kingdom

## Abstract

**Background:**

Although the use of microarray technology has seen exponential growth, analysis of microarray data remains a challenge to many investigators. One difficulty lies in the interpretation of a list of differentially expressed genes, or in how to plan new experiments given that knowledge. Clustering methods can be used to identify groups of genes with similar expression patterns, and genes with unknown function can be provisionally annotated based on the concept of “guilt by association”, where function is tentatively inferred from the known functions of genes with similar expression patterns. These methods frequently suffer from two limitations: (1) visualization usually only gives access to group membership, rather than specific information about nearest neighbors, and (2) the resolution or quality of the relationships are not easily inferred.

**Methodology/Principal Findings:**

We have addressed these issues by improving the precision of similarity detection over that of a single experiment and by creating a tool to visualize tractable association networks: we (1) performed meta-analysis computation of correlation coefficients for all gene pairs in a heterogeneous data set collected from 2,145 publicly available micorarray samples in mouse, (2) filtered the resulting distribution of over 130 million correlation coefficients to build new, more tractable distributions from the strongest correlations, and (3) designed and implemented a new Web based tool (StarNet, http://vanburenlab.medicine.tamhsc.edu/starnet.html) for visualization of sub-networks of the correlation coefficients built according to user specified parameters.

**Conclusions/Significance:**

Correlations were calculated across a heterogeneous collection of publicly available microarray data. Users can access this analysis using a new freely available Web-based application for visualizing tractable correlation networks that are flexibly specified by the user. This new resource enables rapid hypothesis development for transcription regulatory relationships.

## Introduction

Several approaches to microarray data analysis make use of clustering techniques [1]–[4] to suggest functional roles for previously uncharacterized genes. Clustering approaches, however, normally result in a graphical display of groupings that typically lack specific information about the correlation of expression patterns between two selected genes. Thus while group membership can be tentatively established, the topology of the group, or the interactions between its members are not necessarily well elucidated.

Synthesis and visualization of publicly available data remains a challenge for biologists. Available microarray data is thus typically not exploited beyond the scope of the original experiment. Visualization platforms such as Cytoscape [5] or BioTapestry [6] have provided versatile solutions for viewing large networks, including association and interaction networks, but such platforms expect a network provided by the user, and do not learn or reconstruct the networks in and of themselves.

**Figure 1:**
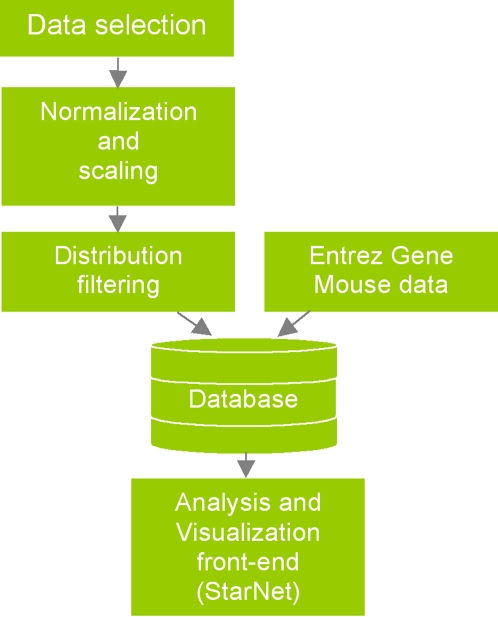
Analysis pipeline. 2145 array samples were selected for the Affymetrix whole genome mouse 430 2.0 array platform. Data were normalized and scaled using justRMALite. The resulting distribution of over 130 million Pearson correlation coefficients was filtered to produce various distributions of the strongest relationships. Correlation data and Entrez Gene annotations were used to populate a new database. StarNet was developed to allow users to make database queries to create and draw correlation networks local to their gene of interest on-the-fly, and to provide supporting information about genes in those networks.

Dynamic Bayesian networks offer a viable approach for the discovery of gene regulatory network topology [7]–[12]. However, these methods are often computationally intensive, heuristic, and limited to the study of small networks usually derived from time series data. Our approach to addressing these issues focuses on visualizing association networks local to a given gene of interest. Using the Affymetrix GeneChip Mouse Genome 430 2.0 Array platform, we **(1)** selected samples from a wide variety of tissues and experimental conditions to build a table of correlation coefficients from all pair-wise comparisons of genes represented on the array, **(2)** selected a subset of those samples in order to examine the differences in network topology which arise in a smaller set of related regulatory states in cardiac tissues and early developmental states, relative to the average regulatory state represented by the full cohort of arrays, **(3)** built a Web based application for user specified network construction and viewing, and **(4)** provide assessment of the resultant networks by drawing networks of known interactions involving the list of genes in the correlation network, and by determining Gene Ontology (GO) [13] annotation terms that are enriched in the correlation network as compared with the entire array platform. All data used in our analyses were retrieved from the Gene Expression Omnibus [14]. **Fig. 1** shows an overview of the project.

We present a user-directed approach to network elucidation, and provide an intuitive Web-based interface (StarNet, http://vanburenlab.medicine.tamhsc.edu/starnet.html) for visual exploration of correlation networks radiating from a selected gene. In short, there are two main parts to the work described here: (1) construction of a database by combining annotations and known interactions from Entrez Gene with our meta-analysis computation of correlation coefficients and data partitioning, and (2) development of a Web-based front end (StarNet) that interrogates the database, constructs networks for visualization, and performs some analyses on those networks to provide a quick assessment of their utility. StarNet results may suggest putative interactions, either in biochemical pathways or transcriptional regulatory networks, thus providing new hypotheses for additional experiments. The results provided by StarNet may also be viewed as the first step in a data analysis pipeline, where the putative networks produced by StarNet, for example, may be studied further using the tools of Bayesian network analysis.

## Methods

### Data Preparation

We selected 2,145 sample hybridizations performed on the Affymetrix GeneChip Mouse Genome 430 2.0 Array which are available from the Gene Expression Omnibus (GEO) [14], [15] for which raw data was available from GEO. Data from these samples, which we have dubbed the “full cohort”, cover a wide range of tissues and experimental conditions. Of these hybridizations, 239 were from experiments related to cardiac development, cardiac tissues in adult mice, or early development (the “cardiac cohort”). A complete list of the experimental datasets used is available at http://www.vanburenlab.tamhsc.edu/starnet_doc.html.

Features on the array were mapped to Entrez Gene [16] IDs using Version 9 of the mapping provided by Dai and colleagues [17]. Their mapping yields 16,297 genes on the array. The arrays within the full and cardiac cohorts were normalized separately, using the justRMALite [18] package within the BioConductor [19] suite of tools. This procedure performs quantile normalization, positive match only adjustment, and Tukey median polish.

Pearson correlation coefficients were calculated for all pairwise comparisons of genes on the array using Octave. This yielded 132,787,956 coefficients for each cohort.

Several subsets of the collection of correlation coefficients were built. First, we selected the 20,000 (20K) largest positive correlation coefficients. This procedure was repeated for 40,000 (40K) and 100,000 (100K) coefficients. The 20K, 40K, and 100K sub-distributions were also formed for the largest negative coefficients. We further considered the union of positive and negative “extreme tails”, for each of the three sizes. This procedure was executed for both the full and cardiac cohorts, yielding a total of 18 different sub-distributions.

To guarantee that each gene on the array is represented in our distributions, a “genecentric” distribution was built. The ten largest positive correlations to each gene were selected, with the proviso that the p-value of the correlation was less than .05. This was repeated for negative correlations, and again the union of positive and negative correlations was considered. This procedure was carried out for both full and cardiac cohorts, thus obtaining an additional six distributions.

We built two further classes of “specialty” distributions, each a variant on the genecentric distribution. First, the genecentric construction was repeated, but constrained to those genes whose GO [13] annotation contains the term “transcription”. Next, the same procedure was repeated for those genes GO-annotated with either of the terms “transcription” or “signal”. This yielded an additional 12 distributions.

### Database

Both sets of correlation coefficients were loaded into a MySQL database, and the partitioning of the set of correlation coefficients was executed using MySQL database calls scripted with Perl.

The database was also populated with Entrez Gene data and Gene Reference Into Function (RIF) files available at NCBI's FTP site (ftp://ftp.ncbi.nlm.nih.gov/gene/; data was retrieved from NCBI on April 26, 2007), which we filtered on their taxonomic ID for mouse entries; Gene RIF interaction data are used in constructing graphs of known interactions in StarNet.

### Network Construction

The network construction algorithms were implemented in Perl. The algorithms allow a variety of choices for defining network topology. For details see the user manual at http://vanburenlab.tamhsc.edu/starnet_doc.html. The relevant parameters (e.g., which type of network to build, which distribution to use) chosen by the user on the submission page are passed to the network building script, and reflected in the script's output.

### Visualization

The CGI script that takes user input from the StarNet submission page and produces the results pages was written in Perl. Graphs are drawn using AT & T's Graphviz package (http://www.graphviz.org). Determination of whether genes in the graph are GO-annotated is achieved by a search against our MySQL database. GO term enrichment in the network is also determined using calls to our MySQL database. The correlation coefficients and counts of array samples, which are needed to determine confidence intervals for correlation coefficients, are written to file by the network construction procedure, and later read by the visualization script. Edges (correlations) are color coded such that darker edges represent stronger correlations. Positive correlations are drawn as shades of blue, and negative as shades of red. Note that the correlation scales were determined on a per network basis. That is, for each network the positive (and/or negative) scales are redrawn, with the scale drawn by equally partitioning between the minimum and maximum positive (and/or negative) correlations within that network. Data used in building graphs of known interactions comes from Gene RIF files available at NCBI's FTP site.

For further details regarding scales, procedures used to build graphs of known interactions, and other details of the visualization script, see the white paper and user manual available at http://vanburenlab.tamhsc.edu/starnet_doc.html.

### Web Site

StarNet takes a user-specified gene as input, as well as the parameters indicated below. Using the distributions described above, a network is then drawn centered about the specified gene. The gene of choice is level 0; those genes to which it is directly connected by correlations from the distribution of choice, and using the graphing methodology of choice, are level 1, etc. Two graphs are produced, one for the cardiac cohort and one for the full cohort. In addition to the correlation graphs we provide **(1)** lists of genes in the graph, hyperlinked to Entrez, **(2)** lists of the edges in the graph, with 95% and 99% confidence intervals for the corresponding correlation coefficients, **(3)** a list of known interactions involving the genes in the graphs, **(4)** a list of genes annotated with the GO term specified by the user, **(5)** a list of GO terms enriched in the graph, hyperlinked to AmiGO, and **(6)** graphs of known interactions involving genes in the networks which StarNet has produced. Additionally, the cardiac and full cohort graphs can be expanded for closer examination, and the nodes in these expanded graphs are hyperlinked to the corresponding entries in Entrez Gene.

To use StarNet (available at http://vanburenlab.tamhsc.edu/starnet.html) the user enters a gene of interest and specifies parameters describing the network to be built and the appearance of the resultant graph. The user submits a gene by entering either the gene's Entrez Gene ID or its gene symbol; a symbol lookup utility is provided. Parameters include the distribution to use (as described above); whether to draw positive, negative, or both positive and negative correlations; the number of levels to draw the network; a parameter specifying network topology; and a GO term for which to search, where those genes annotated with that term will be highlighted in the network and listed. Additionally, there are several parameters to specify aesthetic features of the drawn networks, including alternative color schemes, which may be required by color-blind individuals.

For further details on and explanation of parameter choices, in particular for choices of network topology, see the user manual available at http://vanburenlab.tamhsc.edu/starnet_doc.html.

### Statistical Analysis

Pearson correlation coefficients between genes on the array were computed using Octave (full cohort: n = 2,145, cardiac cohort: n = 239). A two-tailed t-test was used to compute p-values for each coefficient. After using the Fisher z-transformation to normalize the correlation coefficients, confidence intervals in the normalized setting were computed, and the inverse of the Fisher z-transform applied to yield confidence intervals in our original variables.

Enrichment of GO terms was evaluated using the hypergeometric test, following the recommendations of Rivals and colleagues [20] and Gentleman and colleagues [21]. Hypergeometric distribution computations were implemented in Perl. To compute the factorials involved in this distribution, we computed the natural log of the gamma function, using a slight modification of code provided at www.perlmonks.org.

For each of our distributions we computed the mean, standard deviation, and skew. Skew was computed without bias correction. We ran several tests for normality on the distributions: Kolomogorov-Smirnov, Lilliefors, and Jarque-Bera. All were run at the 5% significance level. The Kolmogorov-Smirnov test was run using the sample mean and standard deviation as the parameters for the normal to which to compare our empirical distribution. As the sub-distributions were all found to be non-Normal, we used the Mann-Whitney rank sum test to compare respective sub-distributions from the cardiac and full cohorts. All tests were performed using MATLAB.

## Results

### StarNet

The main contribution of this work is the creation of StarNet, a new freely available Web-based tool that facilitates the reconstruction of transcription regulatory networks via rapid hypothesis development and providing provisional gene groups for focused modeling efforts. A brief description of the tool's features and usage, along with links to supporting documentation, are given in the Methods. Below we describe some features of the sub-distributions that StarNet calls on to construct networks, and we present a sample analysis to show a representative example of StarNet's utility in developing hypotheses about regulatory and pathway interactions with a gene of interest. This demonstration is conducted with *Hand1*, a well-characterized gene, to show that StarNet is capable of generating networks around a gene of interest that are highly consistent with the known characteristics of that gene.

### Distributions and data quality

The genecentric distribution and the distribution of the 100,000 (100K) largest correlations (see Methods) are bimodal in both the cardiac and the full cohort, with one positive mode and one negative mode. Separately examining the positive coefficients and negative coefficients in each sub-distribution reveals that the positive (resp. negative) coefficients are not distributed normally, and are instead skewed to the more extreme values (**Table 1**).

**Table 1:** Sub-distribution statistics.

*Distribution*	*Mean*	*Standard Deviation*	*Skew*	*Number of Genes*
Cardiac 20K Negative	−0.7951	0.0231	−1.2851	2,746
Full 20K Negative	−0.5150	0.0264	−1.6448	3,486
Cardiac 20K Positive	0.9568	0.0117	1.2876	1,534
Full 20K Positive	0.9559	0.0167	0.5900	1,494
Cardiac 40K Negative	−0.7755	0.0259	−1.2907	3,712
Full 40K Negative	−0.4944	0.0282	−1.5856	4,734
Cardiac 40K Positive	0.9458	0.0141	1.0664	2,067
Full 40K Positive	0.9361	0.0239	0.5426	2,265
Cardiac 100K Negative	−0.7457	0.0304	−1.2628	5,122
Full 100K Negative	−0.4648	0.0309	−1.5276	6,670
Cardiac 100K Positive	0.9263	0.0192	0.8767	3,362
Full 100K Positive	0.8957	0.0386	0.5479	4,077
Cardiac Genecentric Negative	−0.5907	0.1342	0.3833	16,297
Full Genecentric Negative	−0.3568	0.1048	0.4748	16,295
Cardiac Genecentric Positive	0.7678	0.1126	−0.2972	16,297
Full Genecentric Positive	0.6856	0.1371	0.1511	16,297

**Figure 2:**
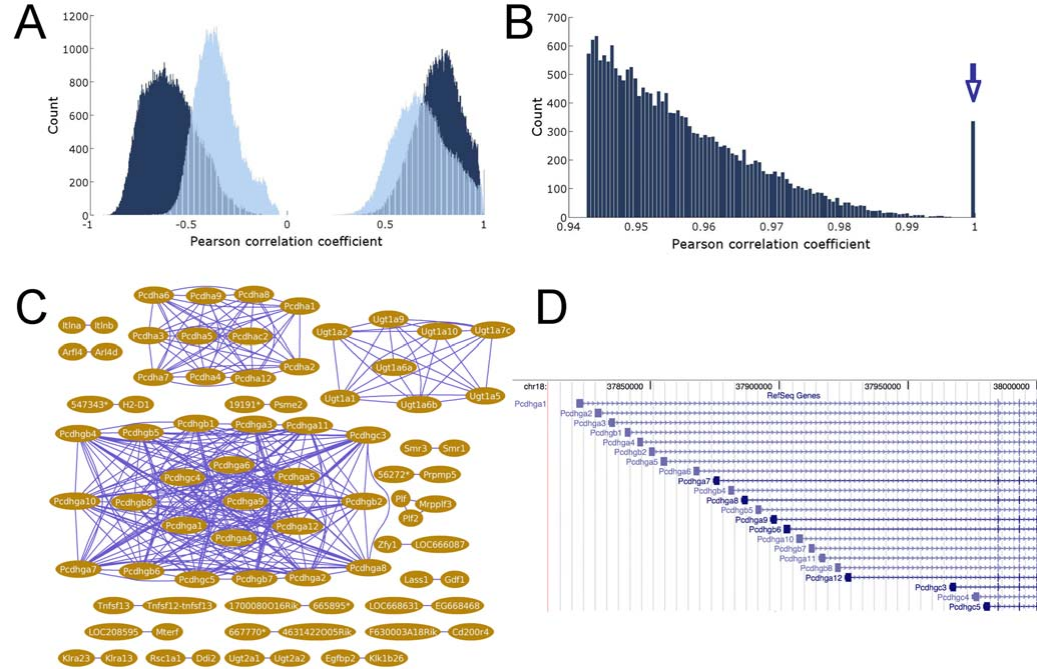
Selected distributions used by StarNet. a: The cardiac cohort (dark blue) and full cohort (light blue) Genecentric distributions. b: The largest positive correlations in the cardiac cohort. The highest 335 of these associations have a Pearson correlation of 1 (to 16 decimal places), and these form a notable spike at the tail of the distribution (arrow). c: The 335 associations indicated in panel b are composed of 80 genes in the 22 groups of genes shown here. d: The genomic structure of the Pcdhgb1 family of genes (drawn with the UCSC Genome Browser).

The mean of the 100K most positive correlations in the cardiac cohort (.9263) is statistically different from the mean of the 100K most positive correlations in the full cohort (.8957) with p<1e-16 (Mann-Whitney rank sum test). The same is true of the means of the 100K most negative correlations in cardiac and full cohorts (p<1e-16), as well as for both positive and negative genecentric distributions (p<1e-16). The correlations in the cardiac cohorts show a general trend of being more extreme than those in the full cohort (**Fig. 2a**).

The full cohort represents a large number of regulatory states, from a variety of tissues, whereas the cardiac cohort represents a relatively fewer number of related regulatory states. The bimodal distributions from the full cohort thus represent an average state consisting of coregulatory and correlative relationships that are relatively weaker on average than those of the cardiac distributions. There are two main factors that contribute to this observed difference between the cardiac and full cohort sub-distributions: **(1)** co-regulation is context-specific, meaning that the transcription activity of two genes may be tightly co-regulated in one tissue or milieu, and weakly co-regulated in others; and **(2)** conditioning the data on a ‘cardiac cohort’, or on any sub-population of tissue types, has a tendency to strengthen measured correlations because of the narrowed range of phenotypes produced by gene activity in those sub-populations. As conditioning increases the average correlation, many gene pairs thus affected that are also highly correlated in other tissues are thus not necessarily specifically corregulated under conditioning. The measured differences between the cardiac and full cohort sub-distributions validate our reasoning for doing separate analyses of the full and cardiac cohorts, and these separate analyses will facilitate future inquiry into the relative contributions of context-specific co-regulation and spurious correlation to the measured differences between the distributions.

At the positive tail of both the cardiac and full cohort highest correlations, there is a spike of 335 correlations equal to 1 (to a precision of 16 decimal places, **Fig. 2b and 2c**). The 80 genes represented by these correlations are distributed into 18 pairs of genes, and one group each of 3 genes, 8 genes, 11 genes and 22 genes, respectively. In seven of the pairs, one of the two gene IDs in the pair was replaced by the other by Entrez Gene, or one of the gene IDs was discontinued. In nine other pairs, as well as the group of three genes, Blast 2 sequences (bl2seq) reveals greater than 90% sequence similarity between the transcripts of the genes within the group, most often at the 3′ ends of both genes, from which Affymetrix's probesets are taken. Two of the pairs cannot be explained using Entrez Gene or overall sequence similarity, but the annotation of Dai and colleagues [17] reveal that the two features in each of these pairs are represented by exactly the same probesets. The remaining three groups are composed of genes that have alternative transcription start sites (families *Pcdhga*, *Ugt1a* and *Pcdha*, respectively). **Fig. 2d** shows the gene structure for the protocadherin family *Pcdhga* represented in the group of twenty-two; each of the genes in our grouping appears in this structure, and all share a common 3′ end. Similar results hold for the group of eight and the group of eleven, although in the latter case there is one gene (*Pcdha1*) in the group that is not in the same locus, but does have 97% sequence similarity with some of the members of the group. Edges in our networks with a correlation of 1 are thus connecting genes that are effectively technical replicates. These results assert the robustness of measuring correlations across different experimental conditions in experiments conducted by different investigators, and demonstrate the generally high reproducibility of measurements made with this Affymetrix platform.

### An example analysis with StarNet: Hand1

As a representative example, below we analyze *Hand1* with the freely accessible Web-based tool StarNet. We selected *Hand1* because its role in cardiac development is well established. The analysis below is intended to illuminate the strengths and weaknesses of StarNet, and is not an attempt to present new results. Analysis was performed with the default settings in StarNet, which includes interrogating the genecentric distribution, and networks are drawn with the highest 5 correlations from the gene of interest (level 1) and the highest 5 correlations for each of those genes (level 2). The genecentric distribution was chosen as a default because this distribution has complete coverage of the array platform. The other parameters were chosen because our testing experience has shown that these parameters produce informative networks that are easily visualized. The StarNet analysis results discussed below may be viewed at http://vanburenlab.tamhsc.edu/Hand1/result.html. Alternatively, these results may also be recreated by typing ‘Hand1’ into the ‘Gene Symbol/Entrez ID’ field of the StarNet interface, and clicking ‘Submit’. With the present implementation, a new analysis takes about two minutes with the default settings in StarNet.

**Figure 3:**
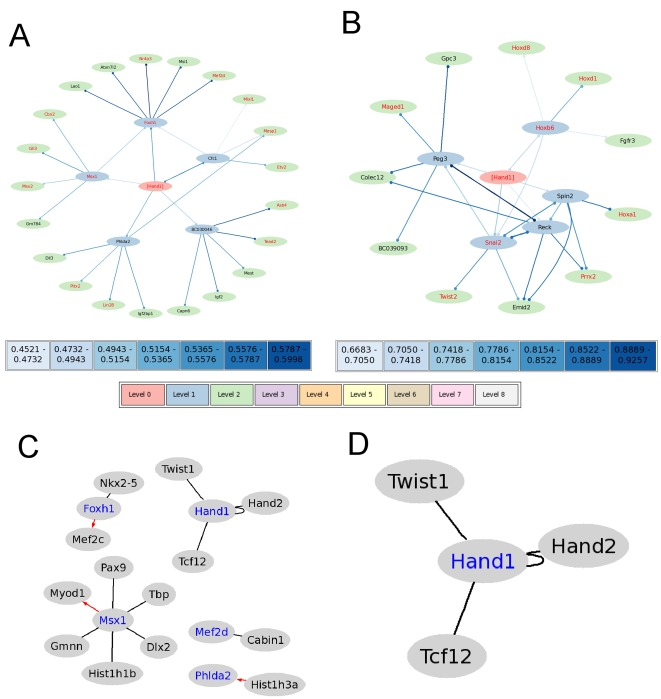
Representative graphical output from StarNet (textual output not shown). a: Full cohort correlation network with *Hand1* as the central node. b: Cardiac cohort correlation network with *Hand1* as the central node. c: Known associations involving genes from a (which appear in blue text). d: Known associations involving genes from B (which appear in blue text). In a and b, red text indicates genes with annotation that include “transcription”, and line color represents correlation strength as indicated by the scale bars. In c and d, black edges represent previously known protein-protein interactions, and red arrows represent previously known protein->DNA interactions. Known interactions for all genes in each correlation network are determined by searching Gene RIF interactions supplied by Entrez Gene.

*Hand1* is implicated in left ventricle formation, and is downregulated in mice lacking *Nkx2-5* [22]. In the cardiac cohort network for *Hand1* drawn with the default parameters in StarNet, we find 7 known DNA-binding genes (*Snai2, Hoxb6, Twist2, Hoxa1, Prrx2, Hoxd1, and Hoxd8*), and 4 genes known to be involved in organ morphogenesis (*Hoxb6, Hoxd1, Hoxd8,* and *Gpc3*), not including *Hand1* itself in either case (**Fig. 3**).

*Cfc1, Msx1, Foxh1, Phlda2*, and *BC030046* all appear as immediate neighbors of *Hand1* in the full cohort network drawn with StarNet using the default parameters (**Fig. 3**). Cfc1 is a Nodal co-receptor, has been shown to control *Lefty* expression in chicks, and has been shown to play a critical role in normal and abnormal cardiovascular development in humans [23]. The homeobox *Msx1* is involved in early neural development and Msx1 interacts with BMP and Smad family members [24]. Foxh1 is known to interact with Nkx2-5, and is essential for anterior heart field development [25]. Deletion of *Phlda2* causes placentomegaly and mice that lack *Hand1* die at embryonic day 8.5 from placental and extra-embryonic abnormalities [22], [26]. *BC030046* is an uncharacterized cDNA sequence derived from preimplantation embryo libraries. *Cfc1, Msx1, Foxh1*, and *Phlda* are thus functionally related to *Hand1*, while the function of embryo-expressed *BC030046* is unknown. The *Hand1* full cohort network is enriched for the GO term, ‘DNA-dependent regulation of transcription’ (*Hand1, Foxh1 , Msx1 , Nr4a3 , Mef2d, Etv2, Mixl1, Lin28, Pitx2, Tead2, Asb4, Gli3, Cbx2, and Msx2*, unadjusted p-value = 0.00000004, hypergeometric test), and most of the genes in the second level of the full cohort network have been implicated in development or heart development (*Dll3, Pitx2, Lin28, Igfbp1, Nr4a3, Msi1, Mef2d, Etv2, Mesp1, Mixl1, Tead2, Igf2, Mest, Gli3, Cbx2* (15 out of 22 genes in the second level)). In particular, *Pitx2* expression is initiated by Nodal, and is left-side expressed in the lateral plate and later in the primordial visceral organs; maintenance of this asymmetrical expression requires *Nkx2-5* [27].

## Discussion

We have noted that known markers for cardiac development appear together more frequently in full cohort networks than in cardiac cohort networks. In the full cohort, where the network is constructed from a more general milieu of associations, genes specifically active in embryonic stages are prominently associated, although it is with relatively smaller correlations than in the cardiac network, on average. Upon examining a finer resolution of associations in the cardiac/development milieu alone (i.e. upon conditioning the measured associations to a narrower range of tissue types), we find that the prominence of correlated genes that are known markers for embryonic or cardiac tissue types in the full cohort networks is frequently displaced by other genes that are more highly correlated within the narrower milieu of the cardiac cohort. Higher correlations between expressed genes are an expected result of conditioning on a narrower field of tissue types. It is also expected that a systematic comparison of the high ranking correlations from each of the cohorts, where networks are built about selected genes implicated in cardiac development, will reveal insights into previously uncharacterized features of cardiac transcriptional regulatory networks.

### Future directions

Correlation, while indicating relationships, does not imply causality. For this reason, the networks built by StarNet should not be viewed as directional, or as indicating that any given gene in the graph is a direct influence on any other. Important relationships are captured by correlation, however, and may thus suggest further experimentation or modeling. Recent work has indicated the utility of correlation as a measure of gene co-expression relationships. For example, Reiss and colleagues [28] discuss co-expression (but emphasize the importance of co-regulation), noting that correlative relationships change depending on the milieu. These issues have also been discussed by other groups [29]–[31], where again the distinction between co-regulation and correlation is made, with co-expression recognized as an important analytical tool. Assertions about causality can be formed using other methodologies such as Bayesian networks and structural equation modeling. Gene lists for networks produced by StarNet can be used as starting material for these methods.

The full cohort and cardiac cohort networks given here as examples of StarNet's analysis are not immediately amenable to quantitative comparisons. One obvious obstacle to comparison is that the networks do not have any nodes in common besides the central node. Many networks drawn with StarNet do have several nodes in common, but the common nodes are frequently a minority of the total network nodes. The networks are constructed with arbitrary cutoffs for the highest correlations with a given node, so many biologically important associations may be missing from a particular network. One approach to comparing these networks would then be to create a ‘super-network’ for each cohort, where all the unique nodes from the full and cardiac network are combined, and a completely connected network is created from the original distribution (full or cardiac) of correlation coefficients. These completely connected networks can be analyzed using the tools of social network analysis [32]. This may be achieved by trimming the completely connected networks according to specified rules. For example, if the correlation between gene X and gene Y is lower than the product of the correlations (X, Z) and (Z, Y), then this suggests that any influence between genes X and Y occurs through the intermediate gene Z. This implies that the edge between X and Y should be trimmed from the network. Upon trimming each network in this manner, it is then straightforward to compute metrics such as *betweeness centrality* or *closeness centrality* and compare them between the two networks. Such quantitative comparisons between networks remain to be developed in future work.

The methodology and algorithms developed to create StarNet may be easily applied to other organisms, other platforms, and any subset of the arrays may be selected as a cohort. Future efforts will expand the utility of StarNet in these areas, and consider comparisons of more than two cohorts.

StarNet adds a useful tool to the repertoire of the biomedical scientist. It is easy to use, and the results are readily interpretable. It can be used in conjunction with the other tools at the biologist's disposal, either as a tool for generating hypotheses for new experimental investigations, or as the first step towards reconstructing and modeling transcriptional regulatory networks.
